# Design of the Xylitol for Adult Caries Trial (X-ACT)

**DOI:** 10.1186/1472-6831-10-22

**Published:** 2010-09-29

**Authors:** James D Bader, Daniel A Shugars, William M Vollmer, Christina M Gullion, Gregg H Gilbert, Bennett T Amaechi, John P Brown

**Affiliations:** 1 Dept. of Operative Dentistry CB#7450, University of North Carolina, Chapel Hill NC 27599-7450 USA; 2 Center for Health Research, 3800 N. Interstate Ave, Portland, OR 97227 USA; 3 University of Alabama at Birmingham School of Dentistry, 1919 7th Ave S, Birmingham, AL 35233 USA; 4 University of Texas Health Sciences Center at San Antonio Dental School, 7703 Floyd Curl Dr., San, Antonio, TX 78229 USA

## Abstract

**Background:**

Dental caries incidence in adults is similar to that in children and adolescents, but few caries preventive agents have been evaluated for effectiveness in adults populations. In addition, dentists direct fewer preventive services to their adult patients. Xylitol, an over-the-counter sweetener, has shown some potential as a caries preventive agent, but the evidence for its effectiveness is not yet conclusive and is based largely on studies in child populations.

**Methods/Design:**

X-ACT is a three-year, multi-center, placebo controlled, double-blind, randomized clinical trial that tests the effects of daily use of xylitol lozenges versus placebo lozenges on the prevention of adult caries. The trial has randomized 691 participants (ages 21-80) to the two arms. The primary outcome is the increment of cavitated lesions.

**Discussion:**

This trial should help resolve the overall issue of the effectiveness of xylitol in preventing caries by contributing evidence with a low risk of bias. Just as importantly, the trial will provide much-needed information about the effectiveness of a promising caries prevention agent in adults. An effective xylitol-based caries prevention intervention would represent an easily disseminated method to extend caries prevention to individuals not receiving caries preventive treatment in the dental office.

**Trial Registration:**

ClinicalTrials.Gov NCT00393055

## Background

Dental caries is a significant problem for adults. Despite a widely held perception that caries is a disease of childhood, it has become clear that the incidence of new lesions in adults is approximately the same as the incidence in adolescents [1]. Dentistry has been slow to recognize and address this problem. Specifically, caries preventive procedures have been infrequently provided to adults in the past [2], although a recent report does suggest that dentists are now more likely to provide such procedures if they identify an adult patient as "caries active"[3].

While it is likely that there are several reasons for the dental profession's inattention to the prevention of caries in adults, lack of evidence about the effectiveness of preventive treatments in adults may be an important factor. Presumably, this lack of evidence contributes to practitioners' reluctance to provide preventive treatment to adults, and may limit purchasers' enthusiasm for coverage for such treatment. The National Institutes of Health Consensus Development Conference on Diagnosis and Management of Dental Caries Throughout Life noted that virtually all of the dental profession's knowledge concerning the effectiveness of caries preventive interventions had come from trials in children and adolescents [4]. The Conference recommended additional caries trials in adults.

In response to that recommendation we designed a randomized clinical trial, the Xylitol for Adult Caries Trial (X-ACT), to test the hypothesis that use of xylitol lozenges will reduce dental caries incidence in caries-active adults. At the time that the trial was designed, three reviews of the effectiveness of xylitol had appeared [5-7]. Although each review found evidence for a xylitol preventive effect, two reviews indicated that the available evidence was not strong enough to permit a firm conclusion of therapeutic effectiveness. All of the reviews indicated that the existing evidence needed to be supplemented by well-designed trials. Because xylitol is already approved as an over-the-counter sweetener, the intervention could be delivered outside the context of a dental office, thus making wide dissemination and implementation feasible. In addition, delivery through candy, gum, or lozenges (mints) would be likely to promote adherence to the preventive regimen. This paper describes the X-ACT protocol.

## Methods/Design

**Figure 1:**
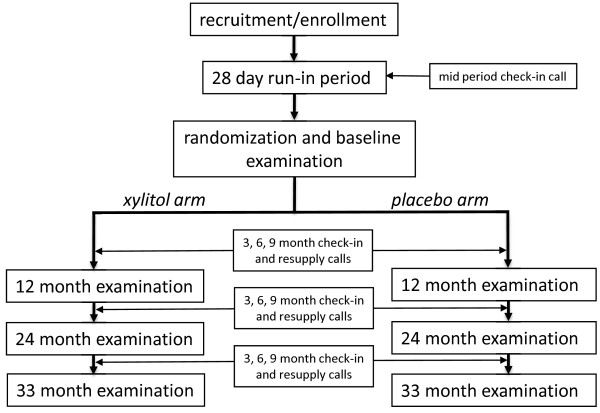
**Trial Design and Participant Contacts**.

X-ACT is a three-year, multi-center, placebo-controlled, double-blind, randomized clinical trial sponsored by the National Institute of Dental and Craniofacial Research (NIDCR) that tests the effects on caries progression of daily use of xylitol lozenges versus placebo lozenges. The trial takes place in three clinical centers, which are located in the dental schools of the University of North Carolina-Chapel Hill, the University of Texas Health Sciences Center-San Antonio, and the University of Alabama-Birmingham. The Data Coordinating Center (DCC) is at the Kaiser Permanente Center for Health Research-Portland, Oregon. A Data and Safety Monitoring Board appointed by NIDCR provides oversight. The study was approved by the Institutional Review Boards at each site and all participants provided written informed consent. An overview of participant flow through the study is shown in Figure 1. Key features are a run-in period before randomization and regular contact of clinical staff with participants throughout the intervention period.

### Study Population

Principal inclusion criteria were age 21-80 and the presence of at least one coronal or root surface cavitated caries lesion (either present at screening or documented in the dental record within the past 12 months). The caries criterion was designed to include participants who were at risk of forming new lesions. Similarly, to ensure an adequate quantity of surfaces at risk, participants were required to have a minimum of 12 teeth with exposed coronal or root surfaces. In addition, participants had to be able to read and comprehend study materials in English, and be able to give informed consent. Exclusion criteria consisted of having more than 10 teeth with caries lesions, having Type IV periodontitis, receiving long-term antibiotic therapy, needing antibiotic prophylaxis prior to dental treatment, having history of head and neck radiation therapy, a history of adverse reaction to either placebo or intervention agent, a serious illness that would interfere with participation, plans to leave the area within the next three years, no telephone, a member of the household already enrolled in the trial, or investigator option.

### Study Treatments

The intervention consisted of consumption of five lozenges daily. We chose lozenges as the delivery method on the basis of a report that lozenges were strongly preferred to gum in a sample of veterans [8] and our own perception that gum would be objectionable to some proportion of potential participants.

Each active lozenge contained 1.0 g of xylitol as a sweetening agent. The placebo lozenge was identical in size and color to the active lozenge but was sweetened with sucralose, which lacks any plausible biologic anti-caries properties. Both the active and placebo lozenges were peppermint flavored. The lozenge used for the run-in period was also sweetened with sucralose, but was spearmint flavored to avoid inadvertent unblinding of participants.

The available literature suggests, but does not demonstrate conclusively, a possible threshold of caries prevention effectiveness around 3 exposures each day with a total daily intake of 3-4 g [9]. Because we were unable to identify a manufacturer capable of compounding both active lozenges containing more than 1 g of xylitol and indistinguishable placebo lozenges, our choice of a 5 lozenge/day regimen represents a compromise between increased assurance of achieving a therapeutic dose and practicality.

Since xylitol has the capacity to act as a laxative in some individuals, participants were asked to gradually increase the number of lozenges they took each day from 1 to 5 during the first 10 days. This "ramping up" was done both during the run-in period and at the initiation of the intervention post randomization.

Lozenges were supplied to participants in containers of 75, with the participant's ID number permanently marked on the container. Seven containers constituted a 3-month supply with some extra lozenges. Participants also received a pocket container capable of holding a day's supply of five lozenges. The active and placebo lozenges were prepared in separate manufacturing runs, and samples of each run were assayed to confirm the xylitol content. The lozenge used during the run-in period was also prepared in a separate run, but was not assayed.

### Study Schedule

Figure 1 summarizes the trial flow from a participant's perspective. The study requires a participant to visit the clinic a minimum of 5 times. Screening was followed as soon as possible by an enrollment visit. The enrollment visit is followed by a placebo run-in period. Adherent, eligible enrollees were randomized at the baseline visit. Participants are then scheduled to return at 12 months, 24 months and 36 months for caries examinations, with quarterly telephone contacts between examination visits. Before the 24-month visits began, the final examination schedule was shifted to 33 months, to adjust for slower than expected completion of enrollment.

### Recruitment, Run-in and Enrollment

Recruitment of potential study participants began in April 2007, and ended in September 2008. Although the primary pool of potential study participants was dental school patients, low yields prompted two sites to expand recruitment efforts to the general public via solicitations at public dental clinics and mass media advertising. All enrollment visits were preceded by a brief conversation by telephone or in person during which eligibility and interest were assessed. Where possible, additional initial screening was accomplished by review of electronic or paper clinical records. All potential study participants attended an enrollment visit where eligibility was verified, the study was described, and requirements of participation were reviewed. Those individuals who indicated a willingness to enroll participated in a four-week run-in period [10] to help identify potential participants who were unable or unwilling to adhere to the study regimen. A call midway through the run-in period was used for data collection (adherence, side effects) and encouragement. Ultimately, 27% of those who entered the run-in period did not continue to randomization [11]. Enrollees received no financial incentives for participation in the run-in period. Financial incentives, in the form of cash payments and travel expense reimbursements, were available for enrollees who continued to the full trial.

### Randomization and Treatment Contacts

Those enrollees who completed the run-in period with adequate self-reported adherence (consumption of more than half of lozenges scheduled during the run-in period) and who indicated a willingness to participate in the full trial attended a baseline visit wherein eligibility criteria were confirmed, and a caries examination was performed. Eligible enrollees were randomized to either the active or placebo arm, additional information was collected, and the initial supply of lozenges was dispensed. Randomization was carried out using a web-based randomization application process. Allocation assignments were stratified by site and age group (≥ 50, < 50 yrs.) in permuted blocks of varying sizes within each stratum. Staff and participants were blinded to treatment assignment. A total of 691 participants were randomized, 92% of the goal of 750.

In the periods between the visits for caries examinations, participants are contacted by telephone on a quarterly basis to assess adherence, side effects, receipt of preventive dental care; to screen for possible serious adverse events; and to arrange for re-supply of lozenges. Participants are asked how many unopened bottles they have on hand, and sufficient bottles for the next three months are dispensed from the participant's master supply at the clinical site and are delivered by courier.

### Caries Examination

Caries are diagnosed visually by calibrated examiners using a CPITN-E probe, a non-magnifying plane mirror; and standard dental operating light and chair. Loupes are used at the discretion of the examiner, but consistently within each examiner. Tooth surfaces are dried for five seconds with an air/water syringe.

The X-ACT examiners use the nomenclature of Pitts and Fyffe [12] to classify the status of each tooth surface; however, the descriptors in X-ACT are a modification of the 2-digit numeric codes in the International Caries Detection and Assessment System II (ICDAS II) [13,14]. ICDAS II has established objective clinical signs that are associated with severity levels of dental caries, verified histologically. The X-ACT taxonomy does not make a number of distinctions that are in the ICDAS II taxonomy. Instead, we have collapsed codes as follows:

• First digit codes: 0 is signified by S(sound); 1, 2 are collapsed into P (pits and fissures sealed surface); 3, 4, 7, 8 are collapsed into F(filled); and 5, 6 are collapsed into C(crowned);

• Second digit codes: 0 is signified by S; 1, 2 are collapsed into a single D_1 _code (uncavitated lesion); 3, 4 are collapsed into a single D_2 _code (cavitated lesion penetrating the enamel); and 5, 6 were collapsed into a single D_3 _code (cavitated lesion penetrating into the dentin).

• Codes 97, 98 were collapsed into M (missing); 96, 99 into Y (unscorable or invisible surface)

For purposes of scoring our outcome measures, calls distinguished by P as the first character are collapsed into the most similar caries status category (see Table 1, e.g., PD_2 _with D_2_), since transition to or from a surface with pits & fissure sealant (P) does not indicate a change in caries status. Calls that signify the need for surgical intervention are treated identically, so the D_2 _and D_3 _calls are collapsed into one category. For our primary outcome, calls that do not require surgical intervention (S, D_1_) are collapsed into one category. Finally, in contrast to ICDAS II, examiners make only one diagnostic judgment per tooth surface. Each central, lateral, and canine tooth is deemed to have five coronal (including the incisal) surfaces and four root surfaces.

**Table 1:** Combined D_2_FS increment: Scoring weight of changes in tooth surface for primary outcome variable

		**T**_**1**_**: Second call**							
**T_0_: First call**		**S [P D**_**1 **_**PD**_**1**_**]**	**D**_**2 **_**[D**_**3 **_**PD**_**2 **_**PD**_**3**_**]**	**F [FD**_**1**_**]**	**FD**_**2 **_**[FD**_**3**_**]**	**C [CD**_**1**_**]**	**CD**_**2 **_**[CD**_**3**_**]**	**Y**	**M**
	
	**S [P D_1 _PD_1_]**	0	1	1	1	0	0	0	0
	
	**D_2 _[D_3 _PD_2 _PD_3_]**	**-1**	0	0	0	0	0	0	0
	
	**F [FD_1_]**	**-1**	**0**	0	1	0	0	0	0
	
	**FD_2 _[FD_3_]**	**-1**	**-1**	0	0	0	0	0	0
	
	**C [CD_1_]**	**-1**	**0**	0	1	0	1	0	0
	
	**CD_2 _[CD_3_]**	**-1**	**-1**	**-1**	0	0	0	0	0
	
	**Y**	0	0	0	0	0	0	0	0
	
	**M**	0	0	0	0	0	0	0	0

S = sound

P = pit and fissure sealant

D_1 _= non-cavitated caries lesion

D_2 _= cavitated enamel caries lesion

D_3 _= caries lesion penetrating into dentin

F = filled

C = crowned

A primary examiner at each clinical center is scheduled to complete virtually all examinations. A back-up examiner can perform examinations in the event that the primary examiner is unavailable. A recorder is present for all caries examinations. Primary and back-up examiners and recorders from all three clinical centers participated in a four-day calibration session with a gold standard examiner and were certified by the DCC prior to the first baseline examinations. They also participate in refresher and reliability measurement sessions prior to the 12-, 24-, and 33-month examinations.

### Study Outcomes

#### Primary Outcome Variable

The primary study outcome is the cumulative D_2 _or Filled Surface (D_2 _FS) increment (root and coronal surfaces combined) cumulated from baseline through the three follow-up examinations. Use of the cumulative, combined D_2 _FS increment measure allows comparison with existing literature and increases confidence that any effect detected represents the prevention cavitation, which has substantial clinical implications for individuals who experience it.

The D_2 _FS increment is computed as the weighted sum, across tooth surfaces, of changes in surface status associated with 64 pre-defined transitions in tooth-surface integrity (Table 1). Our weighting scheme assigned a range of -1, 0, or +1, with transitions to a worse status receiving positive weights and transitions (reversals) to a better status receiving negative weights. No reversals are considered valid for the D_2_FS increment. A transition that reflects no change (e.g., F to F), a change from D_2 _to treated status (F or C), or to or from an unscorable status (Y or M) is scored 0 and hence effectively excluded from analysis. A transition from sound (S) to crowned (C) is given a score of 0 to minimize the influence of non-caries-related treatment on increment counts. Transition from S to CD_2 _is considered implausible given annual examinations. After completion of bootstrapping analyses of a caries increment dataset from another study, we concluded that the optimal way to handle unlikely or implausible transitions is to ignore them (i.e., assign a weight of zero). Hence for our primary analysis the bolded cells in Table 1 will be ignored, although they will be included in an outcome in planned secondary analyses.

#### Secondary Outcome Variable

The cumulative combined D_12_FS increment (Table 2) is a variant of the primary outcome measure that adds transitions to and from non-cavitated caries (i.e., D_1_, PD_1_, FD_1_, CD_1_). This measure is constructed by scoring transitions toward worsening oral health as positive, biologically plausible "reversals" (i.e., remineralization indicated by transition from D_1 _to S) as negative, and no change or implausible/impossible as zero, and then summing over all surfaces examined. Thus, this measure can take negative values. A maximum weight of 2 is assigned to transitions observed between a pair of consecutive examinations that could theoretically be observed as intermediate steps (each contributing an increment) over two to three years of follow-up, so that the cumulative increment could not be greater than any single change. For example, an increment from S to D_2 _is given greater weight on the assumption that it should add the same increment to the cumulative score as two transitions (S to D_1_, then D_1 _to D_2_).

**Table 2:** Combined D_12_FS increment: Scoring weight of transitions in tooth surface for secondary outcome variable.

		**T**_**1**_**: Second call**										
**T**_**0**_**:** **First** **Call**		**S [P]**	**D**_**1 **_**[PD**_**1**_**]**	**D**_**2 **_**[D**_**3 **_**PD**_**2 **_**PD**_**3**_**]**	**F**	**FD**_**1**_	**FD**_**2 **_**[FD**_**3**_**]**	**C**	**CD**_**1**_	**CD**_**2 **_**[CD**_**3**_**]**	**Y**	**M**
	
	**S [P]**	0	1	2	2	2	2	0	0	0	0	0
	
	**D_1 _[PD_1_]**	-1	0	1	1	1	1	1	1	1	0	0
	
	**D_2 _[D_3 _PD_2 _PD_3_]**	**-2**	**-1**	0	0	1	0	0	0	0	0	0
	
	**F**	**-2**	**-1**	**0**	0	1	2	0	1	2	0	0
	
	**FD_1_**	**-2**	**-2**	**-1**	-1	0	1	1	1	1	0	0
	
	**FD_2 _[FD_3_]**	**-2**	**-2**	**-2**	0	**-1**	0	0	0	0	0	0
	
	**C**	**-2**	**-1**	**0**	0	1	2	0	1	2	0	0
	
	**CD_1_**	**-2**	**-2**	**-1**	-1	0	1	-1	0	1	0	0
	
	**CD_2 _[CD_3_]**	**-2**	**-2**	**-2**	**-2**	**-1**	0	0	**-1**	0	0	0
	
	**Y**	0	0	0	0	0	0	0	0	0	0	0
	
	**M**	0	0	0	0	0	0	0	0	0	0	0

S = sound

P = pit and fissure sealant

D_1 _= non-cavitated caries lesion

D_2 _= cavitated enamel caries lesion

D_3 _= caries lesion penetrating into dentin

F = filled

C = crowned

#### Other Study Measures

In addition to the caries examination data, other study data are collected periodically to assist in monitoring participant safety and as measures of risk factors and other mediators of caries incidence. Additional data collected at baseline describe participants' demographic characteristics, self-reported oral hygiene behaviors, fluoride exposure, and exposure to antibiotics. Adherence is measured by self- reported lozenge use at each visit and by the cumulative bottles of lozenges used during the study. Staff also query participants as to receipt of preventive dental care and use of over-the-counter preventive agents since the last contact. Forms used for all data collection are available on the study's public website at http://www.xactstudy.org.

#### Safety Monitoring

A medical history checklist was collected at baseline, and is updated at each subsequent examination visit. Also at every examination visit, participants respond to questions about side effects and possible serious adverse events. Participants are also queried regarding side effects at all between examination telephone calls.

### Quality Control

All clinical center staff were centrally trained and certified in all necessary aspects of study operations, including questionnaire administration and data entry. Initial training occurred prior to the start of randomization, with recertification occurring about annually. The data coordinating center monitors study progress on an ongoing basis and generates regular trial monitoring reports for review by the study's Steering Committee and the Data Safety Monitoring Board (DSMB). The Data Coordinating Center uses a secure, web-based application for data entry and management. This application incorporates real-time error checking and quality assurance at the time of data entry and prompts clinical center staff about potentially erroneous data during data entry. Additional "back-end" checks are performed at the data coordinating center. The data coordinating center maintains an electronic audit trail of all errors and error resolutions. Finally, the DCC conducts annual site visits at each clinical center to check whether data collection, recording, and entry meet standards for quality assurance as defined in Good Clinical Practice (GCP) [15].

### Sample Size

We based our required sample size on a series of large longitudinal cohort studies that permitted estimates of expected combined net D_2_FS increment in the placebo group of the trial. These studies, reviewed by Griffin et al. [16] and Thomson [1] did not include measurement of D_1 _lesions. Although they varied in design, these studies reported sufficient data to calculate reasonable values for three-year untreated incidence rates of root, coronal, and total caries, under the assumption of constant rates over years. These estimated surface increments were 1.4, 2.5 and 3.7 respectively. We estimated the sample size needed, using a range of values (2.3 to 4.9) for the placebo cumulative, combined D_2_FS increment over three years, and assuming that the treatment might result in a 20% reduction, with power set at 80% and one-sided alpha at 0.025. Using PASS 2005 with a Poisson regression model and a large, but plausible overdispersion parameter (3.0, to account for excess variability), and assuming attrition of 10% per year, we obtained a sample size target of 250 at each clinical site (750 total).

### Analysis

#### Missing Data

We expect to have complete data on baseline measures, so missing data are likely to arise only with respect to the annual and final examination visits. Since our analysis approach accounts for time at risk, the primary outcome of combined D_2_FS increment will be imputed from baseline to year 1 only for participants who failed to return for any annual visits, and the time-at-risk will be defined as the target date for the first annual visit. We will use an inclusive model to improve the likelihood of unbiased imputed values [17]. We will multiply impute missing data using data augmentation with Markov Chain Monte Carlo sampling. Five to eight versions of the final analysis dataset will be created and analyzed using identical procedure, followed by combining the results so that variation between imputation versions is incorporated in the standard error and p-value.

#### Primary Outcome Analysis

The primary analyses will be an intent-to-treat (ITT) analysis, on a sample that includes all randomized patients and classifies them according to their assigned treatment group, regardless of their adherence to the protocol. The primary outcome will be analyzed in a negative binomial regression model (using SAS^® ^PROC GENMOD) in which treatment group and clinical center indicators are included as fixed categorical design factors. Planned (*a priori*) covariates are age and age-squared, intensity of preventive regimen, oral hygiene behavior measures, and severity of baseline caries, which is defined as the sum of decayed surfaces at the baseline examination. We will include the natural log of person-years at risk, ln(*t *_i_), as an offset to adjust for length of time since the participant was randomized. Formally, the model is,

ln(μi)=β0 +  Tiβ1+ S1β2 + S2β3 + ci'γ + ln(ti),

or equivalently,

ln(μi/ti) = β0 + Tiβ1 + S1β2 + S2β3++ ci'γ,

where *μ *is the mean observed increment, *μ */t is the incidence of new progressions per year, T is the treatment indicator (0 = placebo, 1 = xylitol), S_1_, S_2 _are clinical site indicators, **c**_i _represents a vector of covariates, and **β **and **γ **represent vectors of parameters to be estimated. The resulting *β*_1 _coefficient thus has the interpretation of the ln(relative risk) for progression for the xylitol compared to placebo lozenges, and the null hypothesis test of no difference between treatment arms is a test of H_0_: *β*_1 _= 0 vs. the alternative hypothesis H_1_: *β*_1 _< 0. The actual fraction of a year's exposure to the lozenges will be used where known, otherwise, the offset will be set to the midpoint of the period between the first examination missed and the last examination attended. In secondary analyses of the primary outcome, we will conduct sensitivity analyses by applying the alternative distributional models (Poisson, mixture models) mentioned above to the same data.

#### Secondary aims

Analyses for three secondary aims are also planned. The first of these analyses tests the primary hypothesis using the secondary outcome variable (cumulative combined D_12_FS increment). As a part of this analysis we will evaluate whether the D_12_FS increment result provides a conclusion for the trial in shorter time. Second, we will compare the intervention's preventive effects on root surfaces as distinct from coronal surfaces. Third, we will examine the impact of lozenge consumption itself on caries prevention in the control subjects, to check whether increased salivary flow might account for differences in the development of new caries, again using the primary and secondary outcomes.

## Discussion

The results of X-ACT should bring some clarity to the controversy over the effectiveness of xylitol as a caries preventive agent. Criticism of available evidence has focused on the design of many of the studies, as well as the heterogeneity of the results [5-7,18]. The lack of placebo controls in many of the trials increases the risk of bias and makes it difficult to disentangle the therapeutic effects of xylitol from the preventive effects of stimulating saliva flow and scrubbing tooth surfaces with gum. In addition, many of the available studies have been evaluated as being at high risk for bias for a variety of other problems, including lack of examiner blinding, lack of examiner calibration, extensive examiner variation, differences at baseline due possibly to classroom or school-level randomization, and high attrition [5,18]. X-ACT employs an active placebo control, is randomized, is adequately powered to detect any caries preventive effect of clinical significance, and adheres to strict standards for good clinical practice. Thus, the results it provides should substantially augment the existing evidence concerning the effectiveness of xylitol.

Further, and perhaps just as importantly, the results will extend this knowledge of effectiveness to adults, for whom so little effectiveness information concerning any caries preventive intervention is currently available. If xylitol lozenges prove to be effective in reducing caries increments in caries-active adults, the intervention would offer such individuals who either do not utilize dental services regularly, or who do not receive caries preventive treatment in the dental office a relatively low-cost, user-controlled caries preventive method.

The inclusion of non-cavitated lesions in the secondary analysis raises some interesting issues with regard to the relative weight given these lesions in the analysis compared to cavitated lesions. We have stated *a priori *our weighting scheme, but plan to analyze variations on this matrix to determine the sensitivity of the increment scores to several of our weighting decisions. Also, because both cavitated and non-cavitated lesions are recorded in this trial, the results should offer insight into the possibility of shortening caries trials by basing the principal analysis on non-cavitated lesion increments. Should a shorter trial be shown to be feasible in retrospect, this will have important implications for future caries trials. The typical three year duration and associated costs of such trials, has contributed to the reduction in such controlled studies in recent decades. Yet the need for this type of unbiased research result for caries preventive agents is undiminished, as discussed above.

## Competing interests

The authors declare that they have no competing interests.

## Authors' contributions

All of the listed authors substantively contributed to the design of the trial, participated in the preparation of the manuscript, and approved the final version of the manuscript.

## Pre-publication history

The pre-publication history for this paper can be accessed here:

http://www.biomedcentral.com/1472-6831/10/22/prepub
